# Perinatal Mental Health Problems in Rural China: The Role of Social Factors

**DOI:** 10.3389/fpsyt.2021.636875

**Published:** 2021-12-07

**Authors:** Qi Jiang, Yian Guo, Evelyn Zhang, Nourya Cohen, Mika Ohtori, Adrian Sun, Sarah-Eve Dill, Manpreet Kaur Singh, Xinshu She, Alexis Medina, Scott D. Rozelle

**Affiliations:** ^1^Stanford Center on China's Economy and Institutions, Freeman Spogli Institute for International Studies, Stanford University, Stanford, CA, United States; ^2^Stanford Pediatric Mood Disorders Program, Stanford University, Stanford, CA, United States; ^3^School of Medicine, Stanford University, Stanford, CA, United States

**Keywords:** perinatal mental health, rural China, depression, anxiety, stress, social support, family conflicts, decision-making power

## Abstract

**Background:** Perinatal mental health is important for the well-being of the mother and child, so the relatively high prevalence of perinatal mental health problems in developing settings poses a pressing concern. However, most studies in these settings focus on the demographic factors associated with mental health problems, with very few examing social factors. Hence, this study examines the prevalence of the depressive, anxiety and stress symptoms among pregnant women and new mothers in rural China, and the associations between these mental health problems and social factors, including decision-making power, family conflicts, and social support.

**Methods:** Cross-sectional data were collected from 1,027 women in their second trimester of pregnancy to 6 months postpartum in four low-income rural counties in Sichuan Province, China. Women were surveyed on symptoms of mental health problems using the Depression, Anxiety, and Stress Scale (DASS-21) and social risk factors. Multivariate logistic regression analyses were conducted to examine social risk factors associated with maternal mental health problems, with results reported as odds ratios (OR) and 95% confidence intervals (CI).

**Results:** Among all respondents, 13% showed symptoms of depression, 18% showed symptoms of anxiety, 9% showed symptoms of stress, and 23% showed symptoms of any mental health problem. Decision-making power was negatively associated with showing symptoms of depression (OR = 0.71, CI: 0.60–0.83, *p* < 0.001) and stress (OR = 0.76, CI: 0.63–0.90, *p* = 0.002). Family conflict was positively associated with depression (OR = 1.53, CI: 1.30–1.81, *p* < 0.001), anxiety (OR = 1.34, CI: 1.15–1.56, *p* < 0.001), and stress (OR = 1.68, CI: 1.41–2.00, *p* < 0.001). In addition, social support was negatively associated with depression (OR = 0.56, CI: 0.46–0.69, *p* < 0.001), anxiety (OR = 0.76, CI: 0.63–0.91, *p* = 0.002), and stress (OR = 0.66, CI: 0.53–0.84, *p* < 0.001). Subgroup analyses revealed that more social risk factors were associated with symptoms of anxiety and stress among new mothers compared to pregnant women.

**Conclusion:** Perinatal mental health problems are relatively prevalent among rural women in China and are strongly associated with social risk factors. Policies and programs should therefore promote individual coping methods, as well as target family and community members to improve the social conditions contributing to mental health problems among rural women.

## Introduction

A growing area of cross-disciplinary research has highlighted the importance of perinatal mental health for the well-being of mothers and children. Multiple studies have shown that perinatal mental health problems are a leading cause of maternal mortality among childbearing women due to higher risks of suicide (1, 2). Poor mental conditions among perinatal women have also been associated with deteriorative obstetric outcomes (3–5). For example, Andersson et al. found that perinatal women with depression and anxiety symptoms had significantly increased nausea and vomiting, prolonged sick leave during the pregnancy and increased number of visits to the obstetrician (3).

As a core part of the early childhood environment, perinatal mental health also plays a crucial role in early childhood development. Research has shown that perinatal mental health problems reduce the effectiveness of child-rearing activities and can contribute to multiple early childhood developmental problems, including impaired cognitive, social, and academic functioning (6, 7). Children of mothers with mental health issues are also two to three times more likely to develop adjustment problems than children of mothers without mental health issues (8). Even as infants, children of depressed mothers are fussier, less responsive to facial and vocal expressions, more inactive, and have elevated levels of stress hormones compared to infants whose mothers are not depressed (9, 10). Literature has also shown that poor mental health is associated with a lack of engaged parenting (7, 11, 12), which may explain why perinatal mental health disorders in caregivers are often associated with developmental delays among infants and young children (11, 13, 14).

Unfortunately, perinatal mental health problems are prevalent in developing settings. In comparison to the 10–13% rate for perinatal depression globally, the prevalence of perinatal depression in developing countries is as high as 20% (15, 16). With the outbreak of the COVID-19, perinatal mental conditions may have become even more prevalent in some settings due to social distancing, disruption of regular antenatal care, and financial difficulties (17). Given the already high prevalence of perinatal mental health problems, along with the documented consequences of mental health problems for the well-being of both mothers and children, a growing number of studies have pointed to perinatal mental health issues as an important area for global public health research (18, 19). In particular, more research is needed to investigate underlying factors that may lead to perinatal mental health problems.

The existing literature describes several demographic and medical risk factors associated with maternal mental health problems in low- and middle-income countries (LMICs). Low levels of family wealth and low maternal education have both been associated with higher rates of perinatal mental health problems (20, 21). Additionally, infant gender (in countries with a preference for males) and parental migration status (in countries where parents leave their children behind to out-migrate for work) may also be related to the mental health of women in LMICs (22, 23). Perinatal health problems such as preterm birth or pregnancy complications also have been shown to be associated with higher rates of mental health problems (24–27).

In comparison to the literature on demographic risk factors, however, fewer studies have examined the role of social risk factors in perinatal mental health. The studies that do exist, however, have identified significant links between social factors and maternal mental health. A study conducted in urban China, for example, found that mothers who receive more social support from family, friends or spouses tended to be at lower risk for stress or depression (27). Another study from rural Pakistan found associations between positive mental health outcomes and maternal autonomy and decision-making power over areas such as finances (14). The same study also found that relationship problems with the spouse or family were associated with worse mental health in mothers (14).

China is the largest developing country in the world, accounting for 15% of the global population. Despite this, little is known about how social factors shape perinatal mental health, especially in rural areas of China, which is home to over half of the country's population. The few studies that have examined mental health issues among rural women and caregivers in China found rates of depression between 23 and 28% and rates of anxiety between 21 and 33% (12, 28). There is also evidence that poor mental health among mothers in rural areas of China is associated with lower levels of interactive parenting and lower levels of cognitive skills among children (12), pointing to long-term consequences of poor mental health beyond the mother's welfare. Unfortunately, no studies to date have examined the association between perinatal mental health and social risk factors in rural China, leaving a considerable knowledge gap.

The overall goal of this study is to investigate the prevalence and risk factors associated with perinatal mental health problems in rural China. Specifically, we pursue two objectives. First, we describe the prevalence of perinatal mental health problems (depression, anxiety and stress) among pregnant women and new mothers in rural China. Second, we identify associations between demographic and social risk factors and symptoms of perinatal mental health problems.

## Methods

This study was conducted in four nationally-designated rural poverty counties in Nanchong prefecture, Sichuan Province. In terms of GDP per capita, Sichuan province ranks 16th out of China's 31 provinces in 2020 (29), and can be considered a middle-income province. Nanchong prefecture, however, ranks relatively low in GDP per capita in Sichuan Province (15 out of 21), and the four study counties in Nanchong have been nationally-designated as poverty-stricken counties (30). The four rural counties are also majority Han ethnicity, the ethnicity that makes up 95% of China's population (31). Therefore, the study area can be considered relatively representative of poor, Han-majority rural areas in the typical Chinese province.

### Sampling

The research team followed a three-step sample selection protocol. First, of the nine counties in Nanchong Prefecture, four nationally-designated poverty counties were selected for sampling. Second, within the sample counties, the research team selected sample townships. The sampling frame excluded non-rural townships and townships with fewer than 10,000 people. Of the remaining townships, 20 townships per county were randomly included in the study, totaling 80 townships.

Third, the research team selected sample households and participants. A list of all households in each sample township with pregnant women beyond their second trimester or infants under 6 months was obtained from the local county-level Maternal and Child Hospital. The research team aimed to recruit 25 eligible households per township. If the township had fewer than 25 eligible households, the sampling frame was expanded to include villages up to 60 mins away from the township. Following this strategy, 1,296 households were sampled. The majority of households in the sample had only one eligible participant (pregnant women or infant). In households with more than one eligible participant, one participant was selected at random. For the purposes of this study, 269 households with infants who were not primarily cared for by their birth mother were excluded. In our final analysis, we use cross-sectional data from 1,027 women, including 309 pregnant women and 718 new mothers.

### Data Collection

Data were collected in November and December, 2019. Trained survey enumerators recruited from public health and medical programs at local universities administered one-on-one survey interviews with participants. All enumerators were native Mandarin speakers and used Mandarin during the training and surveys. Enumerators were supervised by members of the research team during the survey. The survey included three blocks: perinatal mental health (depression, anxiety, and stress), social risk factors (decision-making power, family conflict, and perceived social support), as well as demographic risk factors (characteristics of women, families, and infants).

#### Perinatal Mental Health

To measure the mental health of sample women, enumerators administered the Depression, Anxiety, and Stress Scale-21 (DASS-21), a 21-item short-form version of the DASS-42. DASS-21 was created by Lovibond and Lovibond, and has been validated in China (32, 33). Participants were given a list of 21 statements (7 each for the depression, anxiety and stress subscales) and asked to rank how much the statement applied to them in the past week using a Likert-type scale from 0 = “never” to 3 = “almost always.” Following DASS-21 scoring guidelines, scores for the depression, anxiety and stress subscales were calculated by summing all responses for a given subscale and multiplying the sum by 2. The possible scores for each subscale therefore range from 0 to 42. It is important to note that the resulting score is not a clinical diagnosis but rather a measure of the severity of depression, anxiety, or stress symptoms. The DASS-21 manual also assigns cutoff scores for each subscale, which indicate a relatively high severity of symptoms. Following the DASS-21 manual, women are considered symptomatic of a mental health issue if they scored above 9 on the depression subscale, above 7 for the anxiety subscale, above 14 for the stress subscale. A series of studies examining maternal mental health in rural China to date have used DASS-21 as a measurement for both the symptomatic and severity of mental health issues (12, 28). In our study, the DASS-21 has strong reliability among participants, with a Cronbach's α of 0.824 for the depression subscale, 0.719 for the anxiety subscale, and 0.815 for the stress subscale.

#### Social Risk Factors

The survey assessed three social risk factors: decision-making power, family conflict, and social support. Decision-making power and family conflict were assessed using questions adapted from Shroff et al. (34). and Peterman et al. (35). Women were given eight topics on household decision-making (e.g., family meals, childcare, major purchases, etc.), and were asked to answer (1) whether she had a say in decision-making for this topic, and (2) whether or not there had been a disagreement on this topic in the last month. Supplementary Tables 1, 2 present the itemized responses for decision-making power and family conflict, respectively. Responses were summed to create raw measures of the woman's overall decision-making power and family conflict. Index scores were also created using exploratory factor analysis, which were used in the multivariate analyses.

To measure social support, enumerators administered the Multidimensional Scale of Perceived Social Support (MSPSS), a 12-item subjective assessment of social support created by Zimet et al. and previously validated in China (36–38). Women were given a list of statements that characterized the support they received from family, friends, and significant others (e.g., “I can talk about my problem with my family”) and were asked to rank the statements on a Likert-type scale from 0 (strongly disagree) to 7 (strongly agree). The statements were grouped into three subscales representing family support, friend support, and significant other support, as well as a total social support score (see Supplementary Table 3). The total and subscale scores were calculated by averaging the responses to all questions. The original version of the MSPSS has very good internal reliability, with an α coefficient of 0.88 for the total scale, 0.87 for the family subscale, 0.85 for the friends subscale, and 0.91 for the significant others subscale (38). In addition, its test-retest reliabilities among mothers in our sample were 0.831, 0.849, and 0.801 for the family, friends, and significant others subscales, respectively.

#### Demographic Risk Factors

Data were collected on the individual and family characteristics of sample women, and new mothers were also surveyed on the characteristics of their infants. For individual characteristics, women were asked about their age and education level, whether they were originally from their current town or village, whether they had previously out-migrated for work, whether they planned to out-migrate in the future, whether this was their first pregnancy, and whether they had experienced previous miscarriages. Family characteristics included the husband's age and education, as well as a measure of family assets. To provide a quantifiable estimate of family assets, questions were asked about access to certain household items, such as tap water, computer, internet, a car, and more. A family asset index was then calculated using polychoric principal component analysis (39). Infant characteristics for new mothers were obtained from each infant's birth certificate and included gender, age in months, whether the infant was premature, and whether the infant had low birth weight. New mothers were also asked whether the child was delivered via vaginal birth or by cesarean section (C-section).

### Statistical Analysis

Statistical analyses were performed using STATA 15.1 *P*-values below 0.05 were considered statistically significant. Multivariate logistic regressions of the associations between mental health problems and demographic and social risk factors were conducted among the full sample, as well as among pregnant women and new mothers separately. For the full sample and the pregnant women subgroup, the regressions controlled for the following potential confounders: age, education level, whether the woman was from the village, whether she had previously out-migrated, whether she planned to out-migrate, whether this was her first pregnancy, previous miscarriages, husband's education level, and family asset index score. For new mothers, additional controls were added for infant age, gender, whether the infant was delivered via vaginal birth, whether the infant was premature, and whether the infant had a low birth weight.

## Results

Table 1 presents the summary statistics for the demographic characteristics of respondents. Column 1 reports characteristics of the full sample. The average age of the sample was around 28 years, and about 40% of women had graduated from high school. Just over half (55%) of women were from the village they were living in. A majority of women (78%) had out-migrated for work before, but only 27% planned to out-migrate in the future. About 46% of husbands had completed high school. Among new mothers, the average age of infants was about 3 months, and 55% of sample infants were male. In addition, 44% were born via vaginal birth (the remainder by C-section), 4% of infants were born prematurely, and 3% had low birth weight. Columns 2 and 3 report the characteristics of pregnant women and new mothers, respectively, while Column 4 compares the differences between the two subgroups. The results show no significant differences in the characteristics of pregnant women and new mothers.

**Table 1:** Summary statistics for demographic characteristics among full sample and subgroups.

	**(1)**	**(2)**	**(3)**	**(4)**
	**Full sample**	**Pregnant women**	**New mothers**	**Difference**
	**(*n* = 1,027)**	**(*n* = 309)**	**(*n* = 718)**	**(2)–(3)**
Mother age (years)	27.93 (5.03)	27.89 (5.18)	27.95 (4.97)	−0.06
Mother graduated high school (1 = yes)	0.40 (0.49)	0.39 (0.49)	0.40 (0.49)	−0.01
Mother is from village (1 = yes)	0.55 (0.50)	0.55 (0.50)	0.54 (0.50)	0.01
Mother migrated before (1 = yes)	0.78 (0.42)	0.77 (0.42)	0.78 (0.42)	−0.01
Mother plans to migrate (1 = yes)	0.27 (0.45)	0.24 (0.43)	0.29 (0.45)	−0.05
First pregnancy (1 = yes)	0.22 (0.42)	0.25 (0.43)	0.21 (0.41)	0.04
Previous miscarriage (1 = yes)	0.42 (0.49)	0.39 (0.49)	0.43 (0.49)	−0.04
Husband graduated high school (1 = yes)	0.46 (0.50)	0.46 (0.50)	0.46 (0.50)	0.00
Family asset index (score)	−0.02 (1.02)	0.04 (1.05)	−0.04 (1.00)	0.08
Infant gender (1 = male)	–	–	0.55 (0.50)	–
Infant age (months)	–	–	3.36 (2.10)	–
Vaginal birth (1 = yes)	–	–	0.44 (0.50)	–
Premature (1 = yes)	–	–	0.04 (0.19)	–
Low birth weight (1 = yes)	–	–	0.03 (0.17)	–

*p < 0.05; **p < 0.01; ***p < 0.001. Values are presented as mean (standard deviation). Data source: Authors' survey.

Table 2 reports the measures of social risk factors (decision-making power, family conflict, and perceived social support) for the full sample (Column 1), pregnant women (Column 2) and new mothers (Column 3). For decision-making power and family conflict, both the raw scores (measuring the number of topics for which women reported decision-making power or family conflict) and the index scores (generated using exploratory factor analysis) are presented. The average raw decision-making power score of respondents was 7.20 out of 8, with a standard deviation (SD) of 1.32. The average raw family conflict score was 0.54 out of 8 with a SD of 1.26, and the average social support (MSPSS) score was 5.34 out of 7 with SD of 0.90. When comparing the social risk factors of pregnant women and new mothers (Column 4), the results show a small but statistically significant difference in raw decision-making power scores, with pregnant women reporting greater decision-making power. This difference, however, was not significant when comparing the index scores for decision-making power, and no other differences in social risk factors were found between the two groups.

**Table 2:** Summary statistics for social risk factors among full sample and subgroups.

	**(1)**	**(2)**	**(3)**	**(4)**
	**Full sample**	**Pregnant women**	**New mothers**	**Difference**
	**(*n* = 1027)**	**(*n* = 309)**	**(*n* = 718)**	**(2)–(3)**
Decision-making power raw count (range: 0–8)	7.20 (1.32)	7.33 (1.14)	7.15 (1.39)	0.18*
Decision-making power index score	−0.03 (0.93)	0.02 (0.81)	−0.05 (0.98)	0.07
Family conflict raw count (range: 0–8)	0.54 (1.26)	0.48 (1.16)	0.57 (1.30)	−0.09
Family conflict index score	0.00 (0.91)	−0.04 (0.83)	0.02 (0.94)	−0.06
Social support (MSPSS scale score, range: 0–7)	5.34 (0.90)	5.39 (0.88)	5.31 (0.91)	0.08

*p < 0.05; **p < 0.01; ***p < 0.001. Values are presented as mean (standard deviation). Data source: Authors' survey.

### Prevalence of Perinatal Mental Health Problems

Table 3 presents the prevalence of depression, anxiety, and stress symptoms among all respondents (Column 1), pregnant women (Column 2) and new mothers (Column 3). Column 4 measures the difference between the subgroups of pregnant women and new mothers. Within the full sample, 13% of women showed symptoms of depression, 18% showed symptoms of anxiety, and 9% showed symptoms of stress. Overall, 23% of the women in the study were experiencing symptoms of at least one of the three mental health problems measured. Pregnant women and new mothers showed similar rates of depressive symptoms (14 and 13%, respectively) and stress symptoms (8 and 10%, respectively). However, the prevalence of anxiety among pregnant women was 7 percentage points higher than among new mothers (23 vs. 16%, *p* < 0.01). Pregnant women were also significantly more likely than new mothers to show symptoms of any mental health problem (28 vs. 21%, *p* < 0.05).

**Table 3:** Prevalence of depression, anxiety and stress symptoms among full sample and subgroups.

	**(1)**	**(2)**	**(3)**	**(4)**
	**Full sample (*n* = 1027)**	**Pregnant women (*n* = 309)**	**New mothers (*n* = 718)**	**Difference (2)–(3)**
Symptoms of depression (1 = yes)	0.13 (0.34)	0.14 (0.34)	0.13 (0.34)	0.01
Symptoms of anxiety (1 = yes)	0.18 (0.38)	0.23 (0.42)	0.16 (0.36)	0.07**
Symptoms of stress (1 = yes)	0.09 (0.29)	0.08 (0.27)	0.10 (0.29)	−0.02
Symptoms of any mental health problem (1 = yes)	0.23 (0.42)	0.28 (0.45)	0.21 (0.41)	0.07*

*p < 0.05; **p < 0.01; ***p < 0.001. Participants were considered to have symptoms of a given mental health issue if they scored above 9 for depression, 7 for anxiety, and 14 for stress. Values are presented as mean (standard deviation). Data source: Authors' survey.

### Perinatal Mental Health and Demographic Risk Factors

Supplementary Tables 3–6 present the multivariate logistic regression of associations between demographic risk factors and mental health problems among the full sample, pregnant women, and new mothers, respectively. The results show maternal age and having previously out-migrated were significantly associated with perinatal mental health problems. However, no other demographic risk factors were significantly associated with symptoms of depression, anxiety, or stress. When examining these associations by subgroup, the results show that maternal age and having previously out-migrated were associated to mental health problems among new mothers, but not pregnant women.

### Perinatal Mental Health and Social Risk Factors

Table 4 presents the results of the multivariate logistic regression analysis examining associations between social risk factors and symptoms of perinatal mental health problems. Among the full sample (Panel A), decision-making power, family conflict, and perceived social support were all significantly associated with mental health problems. Women with higher decision-making power index scores (row 1) showed significantly lower odds of having symptoms of depression (OR: 0.71, *p* < 0.001), stress (OR: 0.76, *p* < 0.01), and any mental health problem (OR: 0.76, *p* < 0.001). In contrast, women with higher family conflict index scores (row 2) had significantly higher odds of having symptoms of depression (OR: 1.53, *p* < 0.001), anxiety (OR: 1.34, *p* < 0.001), stress (OR: 1.68, *p* < 0.001), and any mental health problem (OR: 1.49, *p* < 0.001). Finally, perceived social support (row 3) was associated with lower odds of having symptoms of depression (OR: 0.56, *p* < 0.001), anxiety (OR: 0.76, *p* < 0.01), stress (OR: 0.66, *p* < 0.001), and any mental health problem (OR: 0.65, *p* < 0.001).

**Table 4:** Logit regression of associations between social risk factors and perinatal depression, anxiety and stress symptoms.

	**Symptoms of depression**	**Symptoms of anxiety**	**Symptoms of stress**	**Symptoms of any**
**Panel A: Full sample**				
Decision-making power (index score)	0.71*** (0.60–0.83)	0.85 (0.73–1.00)	0.76** (0.63–0.90)	0.76*** (0.65–0.88)
Family conflict (index Score)	1.53*** (1.30–1.81)	1.34*** (1.15–1.56)	1.68*** (1.41–2.00)	1.49*** (1.27–1.75)
Social support (MSPSS total score)	0.56*** (0.46–0.69)	0.76** (0.63–0.91)	0.66*** (0.53–0.84)	0.65*** (0.55–0.77)
**Panel B: Pregnant women**				
Decision-making power (index score)	0.58** (0.41–0.83)	0.84 (0.61–1.16)	0.76 (0.51–1.12)	0.60** (0.42–0.85)
Family conflict (index Score)	1.63** (1.17–2.27)	1.18 (0.88–1.59)	1.63** (1.15–2.32)	1.32 (0.97–1.79)
Social support (MSPSS total score)	0.67* (0.47–0.98)	0.78 (0.57–1.07)	0.84 (0.53–1.33)	0.73* (0.54–1.00)
**Panel C: New Mothers**				
Decision-making power (index score)	0.72** (0.59–0.89)	0.85 (0.70–1.03)	0.75** (0.61–0.92)	0.79* (0.66–0.95)
Family conflict (index score)	1.54*** (1.26–1.88)	1.46*** (1.21–1.76)	1.76*** (1.42–2.18)	1.63*** (1.35–1.97)
Social support (MSPSS total score)	0.50*** (0.39–0.65)	0.72** (0.57–0.91)	0.59*** (0.45–0.79)	0.60*** (0.48–0.74)

*p < 0.05; **p < 0.01; ***p < 0.001. Participants were considered to have symptoms of a given mental health issue if they scored above 9 for depression, 7 for anxiety, and 14 for stress. Values are presented as adjusted OR (95% CI).

*Adjusted regressions include controls for mother's age and education, mother from village, mother migrated before, mother plans to migrate, first pregnancy, previous miscarriage, husband's education, and family asset index. For new mothers, the adjusted regression also controlled for infant gender, age (in months), vaginal birth, prematurity, and low birth weight. Data source: Authors' survey*.

Panels B and C present the social risk factors associated with mental health problems among pregnant women and new mothers, respectively. The results show fewer significant associations between social risk factors and symptoms of mental health problems among pregnant women compared to new mothers. Among pregnant women (Panel B), increased decision-making power (row 4) was associated with significantly lower odds of having symptoms of depression (OR: 0.58, *p* < 0.01) and any mental health problem (OR: 0.60, *p* < 0.01); and family conflict (row 5) was associated with higher odds of depressive symptoms (OR: 1.63, *p* < 0.01) and stress symptoms (OR: 1.63, *p* < 0.01). Social support (row 6) was significantly linked to lower odds of depressive symptoms (OR: 0.67, *p* < 0.05) and symptoms of any mental health problem (OR: 0.73, *p* < 0.05). However, none of the social factors assessed in this study were significantly associated with symptoms of anxiety, and only family conflict was significantly associated with symptoms of stress among pregnant women. In contrast, among new mothers (Panel C), all of the associations were significant except one: decision-making power (row 7) was not significantly associated with symptoms of anxiety.

## Discussion

This study examined perinatal depression, anxiety, and stress symptoms in rural China. Drawing on a large-scale survey of 1,027 pregnant women and new mothers up to 6 months postpartum, we described the prevalence of depression, anxiety, and stress symptoms among the sample. We also examined whether these outcomes were associated with various social risk factors, including decision-making power, family conflicts, and social support.

### Prevalence of Perinatal Mental Health Problems in Rural China

The results of this study show that 13% of pregnant women and new mothers had symptoms of depression, 18% had symptoms of anxiety, 9% had symptoms of stress, and 23% had symptoms of at least one of these mental health problems. The prevalence of perinatal mental health problems in rural China is relatively high compared to the overall global rates among pregnant women (10%) and new mothers (13%) reported by the World Health Organization (16). The results, however, are similar to the findings of a 2012 systematic review of perinatal mental health problems in LMICs, which found that the prevalence of mental health problems was 15.9% among pregnant women and 19.8% among postpartum mothers (15). These results echo the general finding that perinatal mental health problems are more prevalent in LMICs compared to both high-income countries and the overall global prevalence. Importantly, however, the prevalences found in this study are slightly lower than reported in previous studies in rural China (also using the DASS-21 with the same cut-off scores). Previous studies have reported rates of maternal depression between 23 and 28% and rates of anxiety between 21 and 33% (12, 28). While the reason for this variation is unclear, one possible explanation is due to regional/geographical differences in the samples, as past studies were conducted in northwestern rural areas of China, whereas the present study was conducted in rural areas of Sichuan, in southwestern China.

When examining differences between the subgroups, although the prevalence of depression and stress were similar among pregnant women (14 and 8%, respectively) and new mothers (13 and 9%, respectively), the results found a significant disparity in the prevalence of anxiety symptoms among pregnant women (23%) and new mothers (16%). Although this finding stands in contrast to studies in other LMICs such as Pakistan and India, which have found pregnant women to be at lower risk for perinatal mental health problems (14), it is consistent with studies in Nigeria and Thailand which have found pregnant women to generally have higher rates of mental health problems compared to new mothers (40, 41). One explanation may be due to the seasonal timing of data collection for this study. A study of perinatal mental health by Martini et al. (42). found anxiety in pregnant women to be significantly linked to fears about their infant's health, including fear of viral infections. Considering that data for this study were collected in the winter, a season which historically has higher rates of infectious diseases, it is possible that such fears among pregnant women may explain to higher rates of anxiety symptoms. Nevertheless, taken together with the literature, these findings point to a need for more research to compare the prevalence of mental health problems among pregnant women and new mothers and identify underlying causes.

### Perinatal Mental Health and Demographic Risk Factors

Of the demographic risk factors examined in this study, the results found only two significant associations: younger mothers were significantly more likely to have symptoms of all of the mental health problems measured, and mothers who had previously out-migrated were more likely to have symptoms of depression. Subgroup analyses revealed that both variables are only significant among new mothers (and not pregnant women). However, the effect sizes of the associations, while significant, were all relatively small, and no other demographic factors were significantly associated with perinatal mental health problems among the sample.

The absence of associations between other demographic risk factors and perinatal mental health contradicts the findings of previous studies, which have consistently found measures of socioeconomic status, such as education levels and family wealth, to be associated with lower risk for mental health problems (21, 43). One explanation for this discrepancy may be that the sample was selected from nationally-designated poverty counties, meaning that despite some variations in family asset index scores, the respondents were all experiencing similar levels of poverty. Were this sample compared to middle-class urban women in China's cities, it is possible that there would be a more significant relationship between family wealth and mental health. The comparison to urban women would be a complex one, as it would need to account for differences in socioeconomic status, as well as differences in the quality of life and struggles experienced by women in rural and urban areas. Another possible explanation is that the levels of income among households in rural China are higher than other LMICs, given its recent rise to upper-middle income status (44). In either case, these findings indicate that among women in rural China, higher socioeconomic status is not a protective factor in perinatal mental health. The exact reason, however, requires additional research.

Additionally, among new mothers, infant and birth characteristics were not associated with symptoms of mental health problems. Of particular interest, infant gender was not associated with any perinatal mental health problems measured. Although previous studies in China have found that mothers with female infants tend to have higher rates of mental health problems (45, 46), the results of this study are consistent with more recent studies (28), suggesting that the historical cultural preference for males may be shifting.

### Perinatal Mental Health and Social Risk Factors

In contrast to demographic risk factors, social risk factors are strongly and significantly associated with perinatal mental health among both pregnant women and new mothers. For example, women with more decision-making power and social support were 24% and 35% less likely to have symptoms any mental health problems, respectively, whereas women with greater family conflicts were 49% more likely to have symptoms of any mental health problem. These findings agree with a mixed methods study that was conducted in rural northwestern China (a different region from that of our study), which found that lack of social support and lack of agency within the household were two common factors among depressed caregivers (28). The results are also similar to one study in urban China, which found that spousal support contributed to reduced depression symptoms among postpartum mothers (27). These findings are also consistent with the small number of studies in other LMIC settings, which have shown the importance of social factors, particularly within the home, for perinatal mental health (14). There are also a number of studies from high-income countries that have found supportive social dynamics, both within the household and in the community more broadly, to be strongly protective against perinatal mental health problems (42). To date, however, there are relatively few studies examining the relation of social factors to perinatal wellbeing among women in the global south, and more research is needed to better understand factors that shape mental health in poor rural communities.

Although this study is unable to examine the potential mechanisms underlying the associations between social factors and mental health problems among to-be and new mothers, past studies suggest that self-efficacy may be a key mechanism in maternal mental health. Self-efficacy has not only been found to predict mental health symptoms; it has also been identified as a mediator between social factors and mental well-being (47). For example, a longitudinal analysis found that self-efficacy was directly associated with social support and indirectly associated to depression through its relation to social support (48). It is also possible that decision-making power may relate to self-efficacy, however, to date no studies have examined self-efficacy and decision-making power in the context of mental health. Moreover, to the best of our knowledge, however, few studies have examined how self-efficacy shapes the relations between social factors and perinatal mental health in underdeveloped areas. Moreover, to the best of our knowledge, few studies have examined how self-efficacy could shape the relations between social factors and perinatal mental health in underdeveloped areas. Understanding how self-efficacy relates to both social factors and perinatal mental health among rural women in China should be a focus area for future research.

While the associations between social risk factors and depression were similar among all respondents, the associations with anxiety showed differences between pregnant women and new mothers. Interestingly, none of the three social risk factors (decision-making power, family conflict, and perceived social support) were significantly associated with anxiety symptoms among pregnant women, while both family conflict and perceived social support were significantly associated with anxiety symptoms among new mothers. This is striking, considering pregnant women were found to have much higher rates of anxiety symptoms compared with new mothers, and this finding suggests that other factors may be contributing to increased anxiety among pregnant women. One such factor may be the pressure to have a good pregnancy outcome, as past studies have suggested. Further research is needed to identify other possible factors that may be influencing the high rates of anxiety among pregnant women.

### Strengths and Limitations

This study has a number of strengths. To date, the literature on perinatal mental health has focused disproportionately on high income, developed countries, with few studies conducted in developing settings such as rural China. This is one of the first studies to identify risk factors associated with perinatal mental health problems, including mental health problems during pregnancy, among women in rural China. Given that the time around birth is a very unique period in a woman's life, our results positively contribute to the global literature on both mental health and maternal health. Our findings also provide evidence for policymakers and practitioners to design interventions to improve perinatal mental health among women in rural China.

We also acknowledge three limitations. First, due to the cross-sectional nature of the data, we are unable to make temporal or causal inferences regarding the associations between risk factors and perinatal mental health problems. Second, the scales used in this study, although proven to be validated in other settings within China, have not been validated for perinatal women in rural areas of China. In addition, due to the self-report nature of the DASS-21 scale, it is possible that the prevalence of depression, anxiety and stress symptoms are underestimated among women in the sample due to stigma against reporting symptoms of mental health problems. Future research should develop validated scales and collect longitudinal data to better understand the social risk factors that shape perinatal mental health in rural China, and how perinatal mental health impacts other maternal and child health outcomes.

## Conclusions

Among pregnant women and new mothers in rural China, the prevalence of perinatal depression, anxiety, and stress symptoms is relatively high. Pregnant women appear to exhibit higher rates of anxiety symptoms compared to new mothers. Although demographic risk factors are not strongly associated with mental health problems, social risk factors are strongly and significantly associated with depression, anxiety and stress symptoms. These associations are stronger among new mothers compared to pregnant women.

These findings indicate that perinatal mental health is a prevalent problem in rural China that requires greater attention from researchers, policymakers and health practitioners. Policies and programs should be developed to screen pregnant and postpartum women for mental health problems and provide targeted intervention. The strong associations between mental health problems and social risk factors in our sample point to a need for future research examining the extent to which social risk factors may causally contribute to mental health issues, and whether programs targeting in the social environment of women in rural China may reduce the prevalence of depressive, anxiety and stress symptoms during the perinatal period.

## Data Availability Statement

De-identified data will be made available by the corresponding author on reasonable request.

## Ethics Statement

This study received ethical approval from the Stanford University Institutional Review Board (Protocol # 44312). Written informed consent to participate in this study was provided by the participants' legal guardian/next of kin.

## Author Contributions

QJ, YG, EZ, NC, MO, AS, S-ED, and SR contributed to the conceptualization of the study. QJ, YG, EZ, NC, MO, AS, MS, XS, S-ED, AM, and SR contributed to the acquisition, analysis or interpretation of data. YG, EZ, NC, MO, and AS drafted the manuscript. S-ED, MS, XS, AM, and SR made substantial revisions to the manuscript. All authors contributed to the article and approved the submitted version.

## Funding

This research was supported by an unrestricted gift from Enlight Foundation. The funding source played no role in the design of the study, data collection, analysis and interpretation, or writing/editing of the manuscript.

## Conflict of Interest

The authors declare that the research was conducted in the absence of any commercial or financial relationships that could be construed as a potential conflict of interest.

## Publisher's Note

All claims expressed in this article are solely those of the authors and do not necessarily represent those of their affiliated organizations, or those of the publisher, the editors and the reviewers. Any product that may be evaluated in this article, or claim that may be made by its manufacturer, is not guaranteed or endorsed by the publisher.

## Abbreviations

LMICs: low- and middle-income countries
DASS-21: Depression, Anxiety and Stress Scale (short form)
MSPSS: Multidimensional Scale of Perceived Social Support.
