# Supplementary material for: Perinatal Mental Health Problems in Rural China: The Role of Social Factors
Source: Front Psychiatry. 2021 Dec 7;12:636875. doi: 10.3389/fpsyt.2021.636875 (PMC8688533; doi:10.3389/fpsyt.2021.636875)
Supplement: Supplementary file 1 [file Table_1.DOCX]

# Appendix

| **Appendix Table 1. Item responses for decision-making power.** | | |  |  |
| --- | --- | --- | --- | --- |
|  | (1)  Full sample  (n=1294) | (1)  Pregnant Women  (n=336) | | (2)  New Mothers  (n=958) |
| Does woman have decision-making power on what food to buy? (1=yes) | 0.77 | 0.81 | | 0.75 |
|  | (0.42) | (0.39) | | (0.43) |
| Does woman have decision-making power on whether mother should work? (1=yes) | 0.95 | 0.95 | | 0.95 |
|  | (0.21) | (0.22) | | (0.21) |
| Does woman have decision-making power on purchase major goods? (1=yes) | 0.82 | 0.84 | | 0.81 |
|  | (0.38) | (0.37) | | (0.39) |
| Does woman have decision-making power on how to spend money? (1=yes) | 0.86 | 0.87 | | 0.85 |
|  | (0.35) | (0.33) | | (0.35) |
| Does woman have decision-making power on whether to breastfeed? (1=yes) | 0.98 | 0.99 | | 0.98 |
|  | (0.12) | (0.09) | | (0.13) |
| Does woman have decision-making power on how to feed the child? (1=yes) | 0.96 | 0.96 | | 0.96 |
|  | (0.20) | (0.20) | | (0.20) |
| Does woman have decision-making power on what to do if child falls sick? (1=yes) | 0.97 | 0.96 | | 0.97 |
|  | (0.18) | (0.19) | | (0.18) |
| Does woman have decision-making power on how much to spend on healthcare? (1=yes) | 0.93  (0.25) | 0.95  (0.23) | | 0.93  (0.25) |

Notes: Data source: Authors’ survey.

| **Appendix Table 2. Item responses for family conflicts about decision-making.** | |  |  |
| --- | --- | --- | --- |
|  | (1)  Full sample  (n=1225) | (2)  Pregnant women  (n= 350) | (3)  New Mothers  (n=875) |
| Have there been family conflicts in the last month on what food to buy? (1=yes) | 0.12  (0.33) | 0.11  (0.32) | 0.13  (0.33) |
| Have there been family conflicts in the last month on whether mother should work? (1=yes) | 0.1  (0.30) | 0.08  (0.28) | 0.1  (0.31) |
| Have there been family conflicts in the last month on purchase major goods? (1=yes) | 0.07  (0.26) | 0.07  (0.26) | 0.07  (0.26) |
| Have there been family conflicts in the last month on how to spend money? (1=yes) | 0.08  (0.27) | 0.08  (0.27) | 0.08  (0.26) |
| Have there been family conflicts in the last month on whether to breastfeed? (1=yes) | 0.04  (0.21) | 0.04  (0.20) | 0.05  (0.21) |
| Have there been family conflicts in the last month on how to feed the child? (1=yes) | 0.06  (0.24) | 0.05  (0.22) | 0.06  (0.24) |
| Have there been family conflicts in the last month on what to do if child falls sick? (1=yes) | 0.04  (0.19) | 0.03  (0.17) | 0.04  (0.20) |
| Have there been family conflicts in the last month on how much to spend on healthcare? (1=yes) | 0.03  (0.16) | 0.02  (0.14) | 0.03  (0.17) |

Notes: Data source: Authors’ survey.

| **Appendix Table 3. Multidimensional Scale of Perceived Social Support (MSPSS) total and subscale scores** | | | |
| --- | --- | --- | --- |
|  | Total  (n=1228) | Pregnant Women  (n=351) | New Mothers   (n=977) |
| Social Support (MSPSS Total Score) | 5.33  (0.90) | 5.40  (0.88) | 5.30  (0.90) |
| Significant Other Support score | 5.38  (1.05) | 5.46  (1.02) | 5.35  (1.06) |
| Family Support score | 5.50  (1.01) | 5.62  (0.97) | 5.45  (1.03) |
| Friends Support score | 5.09  (1.15) | 5.12  (1.11) | 5.08  (1.16) |

Note: Data source: Author’s survey.

| **Appendix Table 4. Logit regression of correlations between demographic risk factors and perinatal mental health issues (full sample N=1,027).** | | | | |
| --- | --- | --- | --- | --- |
|  | Symptoms of Depression | Symptoms of Anxiety | Symptoms of Stress | Symptoms of Any |
| Mother age (years) | 0.95*  (0.90 - 0.99) | 0.94**  (0.91 - 0.98) | 0.92**  (0.88 - 0.97) | 0.94***  (0.91 - 0.98) |
| Mother graduated high school (1=yes) | 1.16  (0.77 - 1.74) | 1.08  (0.75 - 1.55) | 0.80  (0.49 - 1.31) | 1.02  (0.73 - 1.41) |
| Mother is from village (1=yes) | 0.86  (0.60 - 1.25) | 0.93  (0.67 - 1.28) | 1.20  (0.78 - 1.86) | 0.94  (0.70 - 1.27) |
| Mother migrated before (1=yes) | 1.54  (0.94 - 2.51) | 1.54*  (1.01 - 2.36) | 1.04  (0.61 - 1.76) | 1.29  (0.89 - 1.86) |
| Mother plans to migrate (1=yes) | 1.40  (0.95 - 2.06) | 1.22  (0.86 - 1.74) | 1.54  (0.98 - 2.43) | 1.34  (0.97 - 1.84) |
| First pregnancy (1=yes) | 0.92  (0.55 - 1.54) | 1.18  (0.75 - 1.86) | 0.91  (0.48 - 1.70) | 1.07  (0.71 - 1.62) |
| Previous miscarriage (1=yes) | 1.05  (0.69 - 1.60) | 1.33  (0.92 - 1.94) | 1.41  (0.86 - 2.32) | 1.24  (0.88 - 1.75) |
| Husband graduated high school (1=yes) | 0.86  (0.58 - 1.28) | 0.86  (0.61 - 1.22) | 0.73  (0.45 - 1.17) | 1.00  (0.73 - 1.37) |
| Family asset index (score) | 0.87  (0.73 - 1.04) | 1.00  (0.84 - 1.18) | 0.93  (0.75 - 1.14) | 0.95  (0.82 - 1.11) |
| Note: *p<0.05, **p<0.01, ***p<0.001. Values are presented as adjusted OR (95% CI). Data source: Authors’ survey. | | | | |

| **Appendix Table 5. Logit regression of correlations between demographic risk factors and perinatal mental health issues (pregnant women N=309).** | | | | |
| --- | --- | --- | --- | --- |
|  | Symptoms of Depression | Symptoms of Anxiety | Symptoms of Stress | Symptoms of Any |
| Mother age (years) | 0.95  (0.88 - 1.02) | 0.94  (0.89 - 1.00) | 0.92  (0.83 - 1.02) | 0.95  (0.90 - 1.00) |
| Mother graduated high school (1=yes) | 1.25  (0.59 - 2.65) | 1.16  (0.63 - 2.12) | 1.09  (0.41 - 2.90) | 0.91  (0.51 - 1.62) |
| Mother is from village (1=yes) | 1.12  (0.57 - 2.20) | 0.96  (0.56 - 1.66) | 1.45  (0.61 - 3.42) | 1.07  (0.64 - 1.79) |
| Mother migrated before (1=yes) | 1.03  (0.45 - 2.35) | 1.55  (0.77 - 3.10) | 0.80  (0.30 - 2.18) | 1.12  (0.59 - 2.10) |
| Mother plans to migrate (1=yes) | 1.25  (0.59 - 2.66) | 1.08  (0.58 - 2.01) | 1.10  (0.42 - 2.87) | 1.32  (0.740 - 2.37) |
| First pregnancy (1=yes) | 0.99  (0.39 - 2.48) | 1.16  (0.55 - 2.43) | 0.30  (0.07 - 1.26) | 1.09  (0.54 - 2.21) |
| Previous miscarriage (1=yes) | 0.98  (0.45 - 2.13) | 1.14  (0.61 - 2.15) | 1.10  (0.45 - 2.74) | 1.02  (0.56 - 1.85) |
| Husband graduated high school (1=yes) | 0.53  (0.25 - 1.12) | 0.84  (0.47 - 1.51) | 0.41  (0.15 - 1.09) | 1.10  (0.64 - 1.90) |
| Family asset index (score) | 0.82  (0.60 - 1.11) | 1.03  (0.78 - 1.35) | 0.97  (0.65 - 1.46) | 0.93  (0.73 - 1.20) |
| Note: *p<0.05, **p<0.01, ***p<0.001. Values are presented as adjusted OR (95% CI). Data source: Authors’ survey. | | | | |

| **Appendix Table 6. Logit regression of correlations between demographic risk factors and perinatal mental health issues (new mothers N=718).** | | | | |  |
| --- | --- | --- | --- | --- | --- |
|  | Symptoms of Depression | Symptoms of Anxiety | Symptoms of Stress | Symptoms of Any |  |
| Mother age (years) | 0.94*  (0.89 - 1.00) | 0.94*  (0.89 - 0.99) | 0.92*  (0.86 - 0.98) | 0.94**  (0.90 - 0.98) |  |
| Mother graduated high school (1=yes) | 1.11  (0.68 - 1.81) | 1.06  (0.67 - 1.67) | 0.72  (0.40 - 1.30) | 1.08  (0.72 - 1.61) |  |
| Mother is from village (1=yes) | 0.78  (0.50 - 1.21) | 0.91  (0.60 - 1.38) | 1.20  (0.71 - 2.01) | 0.90  (0.62 - 1.30) |  |
| Mother migrated before (1=yes) | 1.88*  (1.01 - 3.52) | 1.61  (0.93 - 2.79) | 1.10  (0.58 - 2.08) | 1.40  (0.87 - 2.24) |  |
| Mother plans to migrate (1=yes) | 1.44  (0.91 - 2.30) | 1.35  (0.87 - 2.10) | 1.65  (0.97 - 2.81) | 1.40  (0.94 - 2.07) |  |
| First pregnancy (1=yes) | 0.83  (0.44 - 1.57) | 1.17  (0.65 - 2.12) | 1.26  (0.61 - 2.62) | 1.02  (0.60 - 1.72) |  |
| Previous miscarriage (1=yes) | 1.06  (0.64 - 1.77) | 1.53  (0.95 - 2.49) | 1.64  (0.90 - 3.00) | 1.38  (0.90 - 2.11) |  |
| Husband graduated high school (1=yes) | 1.08  (0.66 - 1.75) | 0.90  (0.57 - 1.42) | 0.87  (0.50 - 1.51) | 0.98  (0.65 - 1.46) |  |
| Family asset index (score) | 0.89  (0.71 - 1.11) | 0.98  (0.79 - 1.22) | 0.90  (0.70 - 1.17) | 0.95  (0.79 - 1.16) |  |
| Infant gender (1=male) | 0.90  (0.58 - 1.40) | 0.91  (0.60 - 1.38) | 0.76  (0.46 - 1.27) | 1.00  (0.69 - 1.44) |  |
| Infant age (months) | 0.96  (0.86 - 1.07) | 0.97  (0.87 - 1.07) | 1.01  (0.89 - 1.15) | 0.98  (0.90 - 1.07) |  |
| Vaginal birth (1=yes) | 1.01  (0.65 - 1.59) | 1.21  (0.80 - 1.84) | 1.10  (0.66 - 1.84) | 1.13  (0.78 - 1.63) |  |
| Premature (1=yes) | 1.16  (0.31 - 4.40) | 1.55  (0.50 - 4.76) | 1.86  (0.47 - 7.39) | 1.50  (0.53 - 4.21) |  |
| Low birth weight (1=yes) | 0.72  (0.15 - 3.60) | 1.20  (0.34 - 4.21) | 0.73  (0.14 - 3.86) | 0.83  (0.24 - 2.84) |  |
| Note: *p<0.05, **p<0.01, ***p<0.001. Values are presented as adjusted OR (95% CI). Data source: Authors’ survey. | | | | | |
